# Interferon gamma-induced protein 10 (IP-10) and cardiovascular disease in African Americans

**DOI:** 10.1371/journal.pone.0231013

**Published:** 2020-04-02

**Authors:** Colton Leavitt, Neil A. Zakai, Paul Auer, Mary Cushman, Ethan M. Lange, Emily B. Levitan, Nels Olson, Timothy A. Thornton, Russell P. Tracy, James G. Wilson, Leslie A. Lange, Alex P. Reiner, Laura M. Raffield

**Affiliations:** 1 Division of Biomedical Informatics and Personalized Medicine, School of Medicine University of Colorado, Anschutz Medical Campus, Aurora, CO, United States of America; 2 Department of Medicine, Larner College of Medicine at the University of Vermont, Burlington, VT, United States of America; 3 Department of Pathology & Laboratory Medicine, Larner College of Medicine at the University of Vermont, Burlington, VT, United States of America; 4 Joseph J. Zilber School of Public Health, University of Wisconsin-Milwaukee, Milwaukee, WI, United States of America; 5 Department of Epidemiology, School of Public Health, University of Alabama at Birmingham (UAB), Birmingham, AL, United States of America; 6 Department of Biostatistics, University of Washington, Seattle, WA, United States of America; 7 Department of Biochemistry, Larner College of Medicine at the University of Vermont, Burlington, VT, United States of America; 8 Department of Physiology and Biophysics, University of Mississippi Medical Center, Jackson, MS, United States of America; 9 Department of Epidemiology, University of Washington, Seattle, WA, United States of America; 10 Department of Genetics, University of North Carolina, Chapel Hill, NC, United States of America; Medical University Innsbruck, AUSTRIA

## Abstract

Biomarkers of chronic inflammation (such as C-reactive protein) have long been associated with cardiovascular disease and mortality; however, biomarkers involved in antiviral cytokine induction and adaptive immune system activation remain largely unexamined. We hypothesized the cytokine interferon gamma inducible protein 10 (IP-10) would be associated with clinical and subclinical cardiovascular disease and all-cause mortality in African Americans. We assessed these associations in the Jackson Heart Study (JHS) cohort and the REasons for Geographic and Racial Differences in Stroke (REGARDS) study. There was a modest association of IP-10 with higher odds of left ventricular hypertrophy (OR = 1.20 (95% confidence interval (CI) 1.03, 1.41) per standard deviation (SD) higher natural log-transformed IP-10 in JHS). We did not observe associations with ankle brachial index, intima-media thickness, or arterial calcification. Each SD higher increment of ln-transformed IP-10 concentration was associated with incident heart failure (hazard ratio (HR) 1.26; 95% CI 1.11, 1.42, p = 4x10^-4^) in JHS, and with overall mortality in both JHS (HR 1.12 per SD, 95% CI 1.03, 1.21, p = 7.5x10^-3^) and REGARDS (HR 1.31 per SD, 95% CI 1.10, 1.55, p = 2.0 x 10^−3^), adjusting for cardiovascular risk factors and C-reactive protein. However, we found no association between IP-10 and stroke or coronary heart disease. These results suggest a role of IP-10 in heart failure and mortality risk independent of C-reactive protein. Further research is needed to investigate how the body’s response to chronic viral infection may mediate heart failure and overall mortality risk in African Americans.

## Introduction

Low-grade chronic inflammation, as characterized by elevated circulating levels of innate immunity biomarkers (e.g., C-reactive protein (CRP), interleukin-6, soluble TNF receptor), has been long recognized as an independent risk factor for numerous aging-related chronic diseases such as atherosclerotic cardiovascular disease (CVD) and heart failure (HF), as well as all-cause mortality. Moreover, the recent CANTOS trial demonstrated that inhibition of the pro-inflammatory cytokine interleukin 1-β, which is closely tied to innate immune system function[1], by canakinumab reduced both the risk of CVD events[2] and lung cancer mortality.[3] While immune dysregulation is a common feature of aging, some immunity-related pathways have not been extensively studied in the context of CVD risk. Chronic viral infections such as HIV[4] and hepatitis C (HCV)[5] are associated with increased coronary artery disease risk independent of traditional risk factors. Such chronic viral infection leads to induction of antiviral cytokines, such as type I and type II interferons (IFN), and may promote low-grade inflammation, immune dysfunction, and hypercoagulability.[6, 7] IFN signaling induces a network of interferon-inducible genes, which orchestrate both innate and adaptive immunity in defense against viruses.[8] While IFN is not readily measurable in plasma, interferon gamma-inducible protein 10 (IP-10, also known as C-X-C motif chemokine 10 or CXCL10) may serve as a surrogate chemokine marker for chronic activation of the IFN-1 pathway, distinct from the acute inflammation cascade which is marked by chemokines such as IL-6 and IL-8.[9] Differences in rates of chronic viral infections (including hepatitis B (HBV)[10] and HCV[11]) among African American (AA) populations, as well as known racial disparities in other chronic inflammation measures,[12] may make biomarkers of innate immunity like IP-10 of particular interest in CVD risk assessment in AAs.

IP-10 is secreted by several cell types including T lymphocytes, neutrophils, endothelial cells, monocytes, and fibroblasts. Along with other IFN-induced chemokines (CXCL9/MIG and CXCL11/I-TAC), it can elicit diverse effects in different cell types by binding to a common chemokine receptor, CXCR3.[13, 14] These effects include attraction of T cells and monocytes, regulation of angiogenesis, and differentiation of naive T cells to pro-inflammatory T helper 1 cells.[15, 16] The role of IP-10 in both inflammation and angiogenesis and its involvement in CVD pathogenesis in humans and murine models of atherosclerosis[17, 18] also make it an attractive CVD biomarker for further study. In populations of European origin, IP-10 was associated with incident coronary heart disease (CHD),[19] hypertension,[20] and symptomatic HF,[21] though these associations were not always robust to adjustment for CVD risk factors.[19] However, most studies to date were relatively small, and did not include AA populations who have a higher risk of CVD compared to non-Hispanic whites.[22]

Here, we examined whether higher IP-10 was associated with CVD risk factors, subclinical CVD, and incident events in two prospective cohort studies, Jackson Heart Study (JHS) and Reasons for Geographical and Racial Differences in Stroke (REGARDS).

## Methods

### Study populations

**JHS** recruited 5,306 AA participants in 2000–2004 from urban and rural areas of the three counties (Hinds, Madison and Rankin) comprising the Jackson, Mississippi metropolitan area. Recruitment was limited to non-institutionalized adults who self-identified as AA. Participants were recruited in the following four ways: (1) randomly sampling households from a commercial list, (2) a structured volunteer sample designed to mirror the eligible population, (3) current enrollment in the Atherosclerosis Risk in Communities (ARIC) study, and (4) a nested family cohort. Unrelated participants were between 35 and 84 years old, while members of the family cohort were ≥21 years old at baseline. A range of measures including health behaviors, medication use, anthropometry, blood pressure, kidney function and diabetes, and CVD biomarkers were assessed at baseline.[23, 24] Diabetes was defined as fasting glucose ≥ 126 mg/dL, HbA1c ≥ 6.5%, or self-reported use of a diabetes medication within 2 weeks prior to the clinical visit. Hypertension was defined as blood pressure > 140/90 mmHg or use of blood pressure lowering medication. Serum CRP assays were performed at visit 1 using an immunoturbidimetric CRP-Latex assay from Kamiya Biomedical Company using a Hitachi 911 analyzer.[25] Plasma B-type natriuretic peptide (BNP) was assessed at visit 1 using a chemiluminescent immunoassay as previously described.[26] Serum creatinine was measured using the Jaffe method and calibrated to measurements traceable to isotope dilution mass spectrometry (IDMS).[27] Estimated glomerular filtration rate (eGFR) was calculated from serum concentration of creatinine measured at baseline using the Chronic Kidney Disease Epidemiology Collaboration equation.[28] LDL was calculated using the Friedewald equation in JHS and REGARDS, with individuals with triglyceride values ≥400 mg/dl excluded.

Subclinical CVD was assessed using computed tomography (CT), carotid ultrasound, and echocardiography with imaging data collection, reading, and quality control previously described.[23, 29, 30] Briefly, carotid intima media thickness was quantified using B-mode ultrasonography as the maximum likelihood estimate of average right and left common carotid intima-media thickness.[31] Left ventricular mass index was defined as left ventricular mass calculated using the Devereux et al.[32] formula divided by body surface area. Left ventricular hypertrophy (LVH) was defined as left ventricular mass index >51. Ankle brachial index (ABI) was defined as the ratio of systolic blood pressure (SBP) of the posterior tibial artery to that of the brachial artery using Doppler ultrasound.[33] Cardiovascular imaging for coronary and aortic calcification was conducted at Exam 2, a median of 4.4 years after Exam 1. Agatston scores[34] were used to quantify calcified plaque. Heart and lower abdomen imaging was performed using a Lightspeed 16 Pro 16-channel multidetector system equipped with cardiac gating (GE Healthcare, Milwaukee, WI), using standard protocols developed for the Multi-Ethnic Study of Atherosclerosis (MESA) and Coronary Artery Risk Development in Young Adults (CARDIA) studies.[35] Mortality in JHS was assessed through 12/31/2016 (median follow-up 13.9 years). Incident CVD events were adjudicated through 12/31/2014 (median follow-up 11.8 years for stroke and CHD, 10.0 years for HF) using data from annual follow-up and medical record abstraction by independent reviewers using standardized diagnostic criteria adapted from ARIC.[36–38] Individuals with a prior history of stroke or CHD or who did not consent to medical record abstraction were excluded from incident event analyses. CHD included fatal CHD and myocardial infarction. HF hospitalizations were not adjudicated from the beginning of the study; rather follow-up data was used to determine HF hospitalization status on 01/01/2005. For participants without HF hospitalizations from visit 1 to the beginning of 2005 and without a self-reported history of HF, incident HF events were assessed, with hospitalization events identified through annual follow-up telephone interviews and compared with hospital discharge lists and death certificates by independent reviewers.

**REGARDS** is an observational, longitudinal study of 30,239 AA and white men and women aged ≥45 years designed to assess the reasons for excess stroke mortality in the Southeastern U.S. and among AAs compared to whites.[39] Participants were randomly sampled and recruited across the U.S. between January 2003 and October 2007, aiming to recruit a cohort including approximately equivalent numbers of males and females and whites and AAs, as well as overrepresenting the high risk “Stroke Belt” and “Stroke Buckle” regions in the Southeastern United States. After a telephone interview, an in-home physical exam was completed to assess risk factors including blood pressure and cholesterol levels. Health behaviors, medication use, anthropometry, diabetes, and select CVD biomarkers such as serum creatinine and CRP were measured at baseline as previously reported.[39, 40] Hypertension was defined as SBP≥140 or diastolic blood pressure (DBP)≥90 or self-reported current medication use to control blood pressure. Diabetes was defined by self-report due to greater missingness for a variable based on medication and fasting glucose status. Only AA REGARDS participants were included in this analysis.

Stroke and CHD event ascertainment in the REGARDS cohort has been described previously.[41, 42] Participants or their proxies were contacted every 6 months to ascertain stroke or stroke symptoms and potential CHD hospitalizations. We harnessed the power of the nested case-cohort study in REGARDS[43] and measured IP-10 in all stroke and CHD cases through 12/31/2014 plus an age- and sex- stratified cohort random sample (median follow-up 6.2 years stroke, 5.6 years CHD). This case-cohort design in REGARDS has been used in a number of prior studies to cost-effectively assess the association of biomarkers with stroke and CHD risk (for example [43, 44]). For mortality analyses, individuals were censored at death or date of last known follow-up through 2017 (median follow-up 9.3 years). For CHD, we analyzed a hard CHD variable (definite or probable myocardial infarction or acute CHD death) due to differences in revascularization and other procedure rates by race/ethnicity. Associations with HF could not be adequately assessed in the REGARDS cohort random sample due to a very small number of cases (n = 24).

All study protocols for JHS and REGARDS were approved by institutional review boards at the University of Mississippi Medical Center and by all participating REGARDS institutions (including University of Alabama at Birmingham) [39], respectively, and all participants gave written informed consent.

### IP-10 Measurement

In JHS, IP-10 was measured in November and December 2017 in all individuals with consent for both genetic and epidemiological analyses (n = 3494, n = 5 samples excluded due to insufficient sample volume). In the REGARDS case cohort study, in AA participants only, IP-10 was measured in September 2018 in 454 stroke cases, 477 CHD cases and 515 participants in the cohort random sample[43] (n = 11 with insufficient or no sample volume).

In both cohorts, IP-10 was measured using the R & D Systems enzyme-linked immunosorbent assay (ELISA) in EDTA plasma at the University of Vermont (detectable range ~ 7–1000 pg/mL). Inter-assay CVs from control samples were ~5% in JHS and ~10% in REGARDS. Eight samples above the limit of detection in REGARDS were set to the upper limit (1000 pg/mL), and one sample below the limit of detection was set to the lower limit of detection (7 pg/mL); no samples were above or below the limit of detection in JHS.

### Statistical analysis

In JHS, cross-sectional associations of IP-10 with baseline participant characteristics and subclinical CVD were assessed using generalized estimating equations for quantitative and binary traits (independent correlation structure within families) in SAS 9.3 to adjust for covariates and to account for familial correlation. In regression models, IP-10 was treated as the predictor after adjusting for age and sex. Quantitative traits included BMI, waist circumference, SBP, DBP, total cholesterol, low-density lipoprotein (LDL) cholesterol, high-density lipoprotein (HDL) cholesterol, triglycerides, CRP, ABI, fasting glucose, common carotid intimal thickness (cIMT), left ventricular mass index, coronary artery calcification (CAC), and abdominal aorta calcification (AAC). Binary traits included smoking status (current versus former/never), hypertension, type 2 diabetes, and presence of LVH. Effect estimates were reported per study specific natural log (ln)-transformed SD unit difference in IP-10. AAC, CAC, left ventricular mass index, cIMT, CRP, triglycerides, and BMI were ln-transformed prior to analysis.

In JHS, Cox proportional hazards models with sandwich-based variance estimation were used to evaluate covariate-adjusted associations of IP-10 with all-cause mortality and incident CVD events. Three models with progressive covariate adjustment were used. Model one was adjusted for age and sex only. Model two was additionally adjusted for established CVD risk factors BMI, blood pressure medication use, diabetes, SBP, total cholesterol, HDL and current smoking status. Model three added CRP, a marker for systemic inflammation. IP-10 was treated as a continuous trait (ln-transformed prior to transformation as a z-score, with estimates presented per SD and divided into quartiles (86, 112, 158 pg/mL quartile boundaries). We also performed an exploratory analysis for HF, adjusting for key risk factors eGFR and BNP separately and in the same model. We also included LVH as a potential mediator of the HF association in these exploratory models. We assessed each quartile in reference to the lowest quartile (quartile treated as a class variable) and for trend across quartiles (treated as a continuous variable). Only individuals with complete covariate data for all models were included in incident event and mortality analyses (n = 3173).

In REGARDS, associations of IP-10 with demographic factors were assessed in SAS 9.3 using linear regression models in the stratified cohort random sample only, adjusting for age and sex. In the case-cohort sample, Cox proportional hazard models were used to calculate the hazard ratios (HRs) for stroke, CHD, and mortality. For CHD and stroke outcomes, robust sandwich estimators were used to calculate 95% confidence intervals (CIs) accounting for sample weighting in the case-cohort study design.[43] Models for stroke excluded participants with baseline stroke, and models for CHD excluded participants with baseline CHD; selected stroke cases were also not included in the CHD models, and selected CHD cases were not included in the stroke models. Standard Cox proportional hazards models were used in the cohort random sample to assess the association of IP-10 with mortality. The same 3 covariate models used in JHS were used in the REGARDS analysis, with the addition of geographic region (Southeast Stroke Belt, Stroke Buckle and the rest of the U.S.[39]) to all models. Associations are reported both per ln-transformed SD unit of IP-10 and by IP-10 quartile with quartile boundaries (96, 131, 177 pg/mL) determined in the cohort random sample accounting for sample weighting.

## Results

### Associations between IP-10 and CVD risk factors

Table 1 displays basic demographic variables and CVD risk factors in JHS and REGARDS. In JHS, mean (SD) IP-10 was 140 (105) pg/mL; in REGARDS it was 169 (133) pg/mL. For ln-transformed IP-10, the JHS mean (SD) was 4.79 (0.50); in REGARDS it was 4.95 (0.56) in the cohort random sample. Analyses were conducted on the ln-transformed scale due to the skewed distribution of IP-10 (S1 Fig). In JHS, Table 2 shows IP-10 was significantly lower in males (0.23 SD (95% CI -0.30, -0.15), corresponding to an average IP-10 of 132 pg/mL in males and 146 pg/mL in females in JHS) and was significantly higher with increasing age (β = 3.41 years per SD increase in ln-IP-10, 95% CI 2.98, 3.85) and adiposity (as assessed by both waist circumference and BMI). Higher IP-10 was associated with higher CRP and triglycerides, and with lower LDL, HDL, and total cholesterol, though with modest β estimates. For example, a SD increase in ln IP-10 was associated with a 2.20 cm (95% CI 1.59, 2.81) increase in waist circumference, and a 1.85 mg/dL decrease in total cholesterol (95% CI -3.30, -0.39). Trends were similar in the smaller REGARDS cohort random sample (Table 2), with REGARDS 95% CIs generally containing JHS β estimates.

**Table 1:** Mean, sample size, median, and interquartile range (IQR) of risk factors analyzed in JHS and REGARDS.

	JHS				REGARDS			
	n	Mean (SD) or Frequency	Median	IQR	n	Mean (SD) or Frequency	Median	IQR
**Age (Years)**	3494	55.58 (12.81)	56.30	19.48	515	67.41 (12.12)	68	20
**Male sex (%)**	3494	37.80			515	50.29		
**BMI (kg/m**^**2**^**)**	3487	31.90 (7.30)	30.64	8.58	508	29.67 (6.28)	28.98	7.59
**Waist Circumference (cm)**	3487	101.19 (16.27)	99	20	512	96.92 (14.64)	96.52	19.05
**Current Smoking (%)**	3464	13.31			514	18.60		
**SBP (mmHg)**	3488	127.37 (16.61)	125.66	20.63	512	131.55 (18.11)	130	21
**DBP (mmHg)**	3488	75.77 (8.75)	75.88	12.45	512	78.15 (10.72)	79	13
**Hypertension (%)**	3494	57.30			512	70.04		
**Type 2 Diabetes (%)**	3492	23.14			511	26.12		
Plasma Glucose* **(mg/dL)**	2592	90.40 (8.95)	90	12	378	94.23 (12.50)	93	15
**Statin Medication Use**† **(%)**	3461	13.98			509	29.08		
**HDL Cholesterol (mg/dL)**	3238	51.64 (14.78)	49	19	508	53.34 (16.60)	50.5	20
**Triglyceride (mg/dL)**	3239	107.55 (82.17)	90	64	511	106.58 (52.54)	96	56
**Total Cholesterol (mg/dL)**	3239	199.20 (40.61)	196	51	512	190.04 (40.53)	187	50
**LDL Cholesterol (mg/dL)**	3206	126.49 (36.94)	125	47	506	114.78 (35.48)	111.5	45.5
**CRP (mg/dL)**	3488	0.53 (0.98)	0.26	0.47	508	0.53 (1.11)	0.24	0.45

* Plasma glucose was obtained from fasting blood samples and was assessed only in those without type 2 diabetes.

† REGARDS data is based on self-report of any lipid lowering medication in those with self-reported lipidemia.

**Table 2:** Age and sex-adjusted associations of IP-10 with risk factors in JHS and REGARDS.

	JHS			REGARDS		
	Beta per 1 SD increase in IP-10	SE	P-value	Beta per 1 SD increase in IP-10	SE	P-value
**Age (Years)**	3.41	0.22	<1.0x10^-4^	3.55	0.50	<1.0x10^-4^
**Male sex (%)**	-0.23	0.04	<1.0x10^-4^	-0.25	0.09	4.9x10^-3^
**BMI (kg/m**^**2**^**)**	0.03	4.0x10^-3^	<1.0x10^-4^	-2.0x10^-3^	0.01	0.84
**Waist Circumference (cm)**	2.20	0.31	<1.0x10^-4^	0.78	0.66	0.24
**Current Smoking (%)**	-0.33	0.07	<1.0x10^-4^	0.02	0.12	0.87
**SBP (mmHg)**	-0.18	0.29	0.53	0.13	0.82	0.88
**DBP (mmHg)**	0.09	0.15	0.55	0.51	0.48	0.30
**Hypertension (%)**	0.08	0.04	0.05	0.04	0.10	0.69
**Type 2 Diabetes (%)**	0.08	0.04	0.07	-0.07	0.11	0.52
**Plasma Glucose (mg/dL)**	0.17	0.18	0.36	1.06	0.64	0.10
Statin Medication Use*(%)	-0.05	0.05	0.29	-0.05	0.10	0.65
**HDL Cholesterol (mg/dL)**	-1.90	0.28	<1.0x10^-4^	-1.73	0.70	0.01
**Triglyceride(mg/dL)**	0.05	0.01	<1.0x10^-4^	0.02	0.02	0.29
**Total Cholesterol (mg/dL)**	-1.85	0.75	0.01	-4.55	1.84	0.01
**LDL Cholesterol (mg/dL)**	-0.89	0.68	0.19	-3.62	1.63	0.03
**CRP (mg/dL)**	0.16	0.03	<1.0x10^-4^	0.06	0.06	0.26

Models are adjusted for age and sex (except for age and sex). BMI, triglycerides, and CRP are natural log transformed prior to analysis. 1 SD IP-10 corresponds to 105 pg/mL in JHS and 133 pg/mL in REGARDS.

IP-10 was not associated with most measures of subclinical CVD in JHS (Table 3), including ABI, cIMT, AAC and CAC (assessed at visit 2). Each SD increment higher ln IP-10 was associated with higher odds of LVH (OR = 1.20 (95% CI 1.03, 1.41 and higher left ventricular mass index (β = 0.02 (95% CI 0.01, 0.03) in ln-transformed left ventricular mass index) (Table 3). LVH based on echocardiography was not available in REGARDS, but trends were similar for LVH by Sokolow-Lyon criteria from baseline electrocardiograms [45] (OR = 1.31, 95% CI 1.02, 1.68).

**Table 3:** Association of subclinical CVD measures with IP-10 in JHS, adjusting for age and sex. Beta values are reported per SD of natural log transformed IP-10,including for binary measures. Mean (SD) values or frequency for subclinical CVD measures is also reported.

Subclinical CVD measure	Beta	SE	P-value	n	Mean (SD) or Frequency	Median	IQR
**Ankle Brachial Index (ABI)**	3.8x10^-3^	3.1x10^-3^	0.22	3101	1.21 (0.17)	1.21	0.19
**Carotid intima media thickness (cIMT)**	4x10^-4^	3.6x10^-3^	0.91	3319	0.73 (0.19)	0.71	0.23
**Left Ventricular Mass Index**	0.02	0.01	2.8x10^-3^	2234	36.31 (9.79)	34.42	11.06
**Coronary Artery Calcium (CAC) Agatston Score**	0.05	0.06	0.36	1939	167.71 (506.81)	0	86.45
**Abdominal Aorto-iliac Calcium (AAC) Agatston Score**	-0.03	0.06	0.62	1938	895.30 (1628.78)	115.07	1077.13
**Left Ventricular Hypertrophy (LVH)**	0.19	0.08	0.02	2234	7.83%		
**Any AAC**	0.02	0.06	0.76	1938	66.36%		
**Any CAC**	0.01	0.05	0.84	1939	48.84%		

*Carotid IMT, left ventricular mass index, AAC and CAC were natural log transformed prior to analysis.

### Association between IP-10 and incident events

There were 559 deaths, 110 strokes, 147 CHD cases and 190 HF cases in individuals with complete covariate data in JHS (Table 4). In REGARDS there were 445 strokes, 466 CHD cases, and 160 deaths in individuals with complete covariate data. The association between IP-10 and mortality in REGARDS was only assessed in the cohort random sample. In the analysis of IP-10 as a continuous variable, each higher SD increment of IP-10 was associated with an increased risk for both death (HR 1.12 per SD 95% CI 1.03, 1.21) and incident HF in JHS (HR 1.26 per SD, 95% CI 1.11, 1.42), adjusting for CVD risk factors and CRP (Model 3). Compared to those in the lowest quartile of IP-10, participants in the highest quartile of IP-10 had a HR of 1.50 (95% CI, 1.15, 1.96) for all-cause mortality and 2.74 (95% CI 1.66, 4.52) for incident HF after risk factor adjustment including CRP (Model 3) (S1 Table). This HF HR in JHS was only modestly attenuated upon adjustment for BNP and eGFR (HR 2.33; 95% CI 1.38, 3.91, S2 Table). Additional inclusion of LVH as a covariate also only modestly attenuated this association (HR 2.72, 95% CI 1.48, 4.99). The association of IP-10 with mortality was also observed in REGARDS (HR 1.31, 95% CI 1.10, 1.55, per SD higher and HR 2.16, 95% CI 1.22, 3.82 comparing the highest to lowest quartiles of IP-10). There was no association between IP-10 and incident CHD or stroke with any model of risk factor adjustment in JHS or REGARDS. Kaplan-Meier plots visualizing all non-case-cohort designed analyses for IP-10 quartiles (no covariate adjustment) are displayed in S2 Fig.

**Table 4:** Association of IP-10 with mortality and incident cardiovascular disease events in JHS and REGARDS.

		JHS			REGARDS		
Model		1	2	3	1	2	3
**Coronary Heart Disease**	**Events/N**	101/2906			466/868		
	**HR**	0.98	0.98	0.97	1.14	1.16	1.15
	**(95% CI)**	(0.80, 1.19)	(0.82, 1.17)	(0.81, 1.16)	(0.97, 1.33)	(0.96, 1.39)	(0.95, 1.38)
	**p-value**	0.81	0.80	0.76	0.11	0.13	0.16
**Stroke**	**Events/N**	110/2991			445/881		
	**HR**	1.05	1.07	1.06	1.15	1.17	1.17
	**(95% CI)**	(0.85, 1.31)	(0.88, 1.31)	(0.88, 1.28)	(0.98, 1.36)	(0.98, 1.40)	(0.97, 1.40)
	**p-value**	0.64	0.49	0.52	0.09	0.09	0.10
**All-Cause Mortality**	**Events/N**	559/3173			160/475		
	**HR**	1.10	1.12	1.12	1.33	1.36	1.31
	**(95% CI)**	(1.01, 1.19)	(1.03, 1.22)	(1.03, 1.21)	(1.13, 1.56)	(1.15, 1.60)	(1.10, 1.55)
	**p-value**	0.02	5.8 x 10^−3^	7.5 x 10^−3^	5 x 10^−4^	4 x 10^−4^	2.0 x 10^−3^
**Heart Failure**	**Events/N**	190/2756					
	**HR**	1.28	1.26	1.26			
	**(95% CI)**	(1.13, 1.45)	(1.11, 1.42)	(1.11, 1.42)			
	**p-value**	1 x 10^−4^	4 x 10^−4^	4 x 10^−4^			

* Hazard ratios (HR) and 95% confidence intervals (CIs) are reported per standard deviation increase in IP-10. Only individuals with complete covariates for all models are included. REGARDS did not have an adequate number of heart failure cases in the case-cohort study for analysis.

† **Model 1:** Adjusted for age, sex (with additional adjustment for region in REGARDS)

‡ **Model 2:** Model 1 + BMI, blood pressure medications, type 2 diabetes, SBP, total cholesterol, HDL cholesterol, current smoking

§ **Model 3:** Model 2 + CRP

To further explore the observed association with mortality, we performed exploratory analyses for cause-specific mortality. Specific information on cause of death was not available in JHS. In REGARDS, of the 160 deaths in the random cohort sample, 158 had an adjudicated cause of death; 56 were from CVD, 45 from cancer, 15 from infection, 19 from dementia, and 23 from other causes (such as end-stage renal disease, infection, or other). Adjusting for age, sex and region, each SD increment higher ln-IP-10 concentration was associated with death from cancer (HR 1.52, 95% CI 1.14, 2.03, p = 0.004), but was not associated with death from dementia (HR 0.72, 95% CI 0.39, 1.34, p = 0.31), infection (HR 1.46, 95% CI 0.81, 2.62, p = 0.21), or overall CVD (HR 1.26, 95% CI 0.97, 1.63, p = 0.08).

## Discussion

We examined the relationship of circulating levels of IP-10, an IFN-dependent chemokine, with CVD outcomes and mortality in two community-based prospective studies of AAs. IP-10 was significantly associated with both HF and all-cause mortality. These associations were independent of traditional CVD risk factors, including CRP. However, there was no association between IP-10 and stroke or CHD. Among the many subclinical disease measures analyzed (arterial calcification, LV mass measures, intima media thickness, and ankle brachial index) only an association with LV mass was identified. This study adds significantly to the limited literature on the role of IP-10 as a CVD risk biomarker in large population-based cohorts, specifically in AAs, and suggests that further study of IP-10 and interferon-induced viral response pathways is warranted in HF in AAs.

We are not aware of other large prospective studies linking IP-10 levels with HF and LVH. In a previous small case-control study of HF, IP-10 and other CXCR3 ligands were associated with HF independently of BNP.[46] There is considerable evidence that T lymphocytes and the adaptive immune system are involved in chronic inflammation associated with both atherosclerotic CVD and HF.[47, 48] IP-10 is a chemoattractant for cardiac infiltration of pro-inflammatory T helper 1 cells and cytotoxic T cells, and was elevated in human and experimental models of atherosclerosis, HF, and cardiac dysfunction and remodeling.[17, 21, 49] In ischemic models of cardiac repair, IP-10 appears to inhibit fibroblast migration and limit cardiac fibrosis, thereby promoting normal myocardial tissue remodeling,[50] possibly through CXCR3-independent signaling pathways.[51] Similarly, IP-10 inhibited fibroblast migration and accumulation in bleomycin-induced pulmonary fibrosis.[52] IP-10 levels are also associated with intrahepatic inflammation and fibrosis in individuals infected with HCV and with other chronic liver diseases.[53, 54] By contrast, in both murine and human models of pressure overload–induced cardiac dysfunction, IP-10 appears to promote CD4^+^ T helper cell heart infiltration and adverse fibrosis and cardiac remodeling in a CXCR3-/LFA-1/ICAM-1-adhesion pathway.[49] Further analysis of the role of IP-10 and its receptor and other IFN-γ-inducible ligands in ischemic vs. non-ischemic HF may shed additional light on the balance of fibrotic and specific T-cell subset-dependent processes involved in cardiac tissue repair.[55] Given the higher incidence of HF and left ventricular dysfunction in AAs,[56] gaining a better understanding of the links between adaptive immunity biomarkers and HF is particularly important.

Our results on the correlates of IP-10 in AAs are consistent with previous results from the large MONICA/KORA Augsburg Case-Cohort Study in Europeans,[19] which had 381 CHD cases and 1977 without CHD. Specifically, IP-10 was positively correlated with several CVD risk factors, including age, BMI, and other inflammatory biomarkers like CRP, but negatively correlated with cholesterol levels, including HDL. However, the sex difference in IP-10 observed in JHS was not observed in MONICA/KORA Augsburg.[19] The magnitude of the effect was similar in REGARDS but was not statistically significant. We also did not observe any evidence for association of IP-10 with hypertension or SBP and DBP levels in contrast to the MONICA/KORA Augsburg study[19] and several other smaller case-control samples,[20, 57] including one case/control study (n = 140 controls, n = 140 untreated hypertension cases) which found that those with systolic and/or diastolic hypertension had an average IP-10 level of 148 pg/mL, while those without had average IP-10 levels of 98 pg/mL (P<0.001).[20] Correlations in the latter study were attenuated but still significant when adjusted for age, BMI, creatinine, glycemia, HDL, LDL and triglycerides.

The lack of association between IP-10 and incident CHD or stroke in JHS and REGARDS can be compared to two prior studies that examined the relationship of IP-10 to incident CVD outcomes. In a cohort of AAs with type 1 diabetes, higher IP-10 was associated with higher risk of incident composite CVD events (defined as CHD, stroke, or peripheral arterial disease) independently of age, BMI, smoking, and mean arterial pressure with an OR = 3.53 (95% CI 1.07, 11.64) comparing the top and bottom quartiles of IP-10.[58] This study was small (n = 320 with only 43 incident CVD events) and only included individuals with type 1 diabetes without complication from infection or systemic inflammatory conditions. However, in the MONICA/KORA Augsburg study, the largest population-based study in European populations for IP-10 with CVD, the association of IP-10 with incident CHD was attenuated to insignificance (third tertile compared to first HR = 1.26 95% CI 0.94–1.69) after adjustment for established CHD risk factors.[19] Our results are essentially concordant with this estimate of small to no effect of IP-10 on CHD risk from the MONICA/KORA Augsburg study. We did note in JHS that there was some evidence of mortality being a competing risk for CHD (p = 0.01) and heart failure (p = 0.02), though not for stroke (p = 0.09).

To our knowledge, this is also the first report of an association between IP-10 and overall mortality in a general population. While biomarkers of innate immunity such as CRP and IL-6 are well-documented risk factors for total mortality,[59, 60] particularly in older adults, fewer studies have examined the relationship of T-cell immune dysregulation and mortality in the general population despite the well-documented association of aging with T-cell immune dysfunction.[61] Both aging and chronic viral infection are characterized by low-level chronic inflammation and immune activation which in turn contribute to the development of age-related diseases, frailty, and mortality. IP-10 is not an acute phase reactant, instead tagging chronic IFN mediated immune responses. Consistent with previous studies[9, 19], in REGARDS IP-10 is only modestly correlated (Spearman correlation ≤0.3) with IL-6, IL-8, and CRP, commonly measured inflammation biomarkers associated with the acute phase response. Chronic viral infections such as Cytomegalovirus, Herpes Simplex Virus and HCV are common in adult populations and can result in altered T-cell immune responses, which may be related to various chronic diseases, frailty, and longevity.[62] Measurement of IP-10 and other type I and II IFN-induced chemokines in large population-based samples of older adults, and comparison of predictive models with other inflammatory markers, may shed further light on the role of T-cell immune response and immune dysfunction in healthy and less healthy aging. Racial disparities in chronic viral infection rates (for example the tenfold excess of chronic HCV infection rates in AAs over 60 years of age versus other race/ethnicity groups[11]) make this field of research particularly important in AA populations.

Our study has several strengths including the evaluation of two large AA cohorts: a community-based sample from Jackson, Mississippi and a nationally sampled cohort, both followed prospectively for incident CVD outcomes and mortality. Some limitations should be noted, including the lack of most subclinical disease measures and the small numbers of HF cases in the REGARDS cohort random sample. HF was also not assessed at baseline in JHS, so incident HF was assessed only from 2005–2014. Additionally, JHS and REGARDS do not have data on viral titers or self-reported diagnoses for common chronic viral infections such as HBV and HCV. IP-10 has been correlated with chronic viral infection in previous studies[63, 64] but we were not able to examine this. Future work should further examine cause of death in larger samples in order to better understand why IP-10 is associated with overall mortality and explore suggestive associations with risk of cancer mortality.

In summary, IP-10 was associated with increased risk of mortality and HF in two population-based cohorts of AAs. This work suggests that further investigation into the potential role of chronic viral infection and T-cell dysregulation in HF risk is warranted in AA populations.

## Supporting information

S1 Fig Distribution of IP-10 (pg/mL) and natural log transformed IP-10 in jackson heart study and the REasons for geographic and racial differences in stroke (REGARDS) study. (PDF)

S2 Fig Kaplan-Meier plots for IP-10 quartiles, unadjusted for covariates. (PDF)

S1 Table Association of IP-10 quartiles with mortality and incident cardiovascular disease events in JHS and REGARDS. Each quartile is reported in reference to quartile 1. Trend across quartiles is also reported.
(PDF)

S2 Table Additional adjustment for the potential confounders kidney function (as assessed using estimated glomerular filtration rate (eGFR) calculated using the CKD-EPI equation) and Brain Natriuretic Peptide (BNP) in the JHS heart failure models, as well as potential mediator left ventricular hypertrophy. (PDF)

## Abbreviations

AAC: Abdominal Aorta Calcification
AA: African American
ARIC: Atherosclerosis Risk in Communities
BNP: B-type natriuretic peptide
CVD: Cardiovascular Disease
cIMT: Common Carotid Intimal Thickness
CAC: Coronary Artery Calcification
CHD: Coronary Heart Disease
DBP: Diastolic blood pressure
eGFR: Estimated glomerular filtration rate
CRP: CRPC-reactive protein
IP-10: Interferon gamma inducible protein 10
IFN: Interferon
JHS: Jackson Heart Study
LVH: Left Ventricular Hypertrophy
LDL: low-density lipoprotein
REGARDS: Reasons for Geographic and Racial Differences in Stroke
SBP: Systolic blood pressure
