# Supplementary material for: Interferon gamma-induced protein 10 (IP-10) and cardiovascular disease in African Americans
Source: PLoS One. 2020 Apr 2;15(4):e0231013. doi: 10.1371/journal.pone.0231013 (PMC7117698; doi:10.1371/journal.pone.0231013)

Supplementary Figure 1: Distribution of IP-10 (pg/mL) and natural log transformed IP-10 in Jackson Heart Study and the REasons for Geographic and Racial Differences in Stroke (REGARDS) study.

a. JHS, IP-10 untransformed values in pg/mL

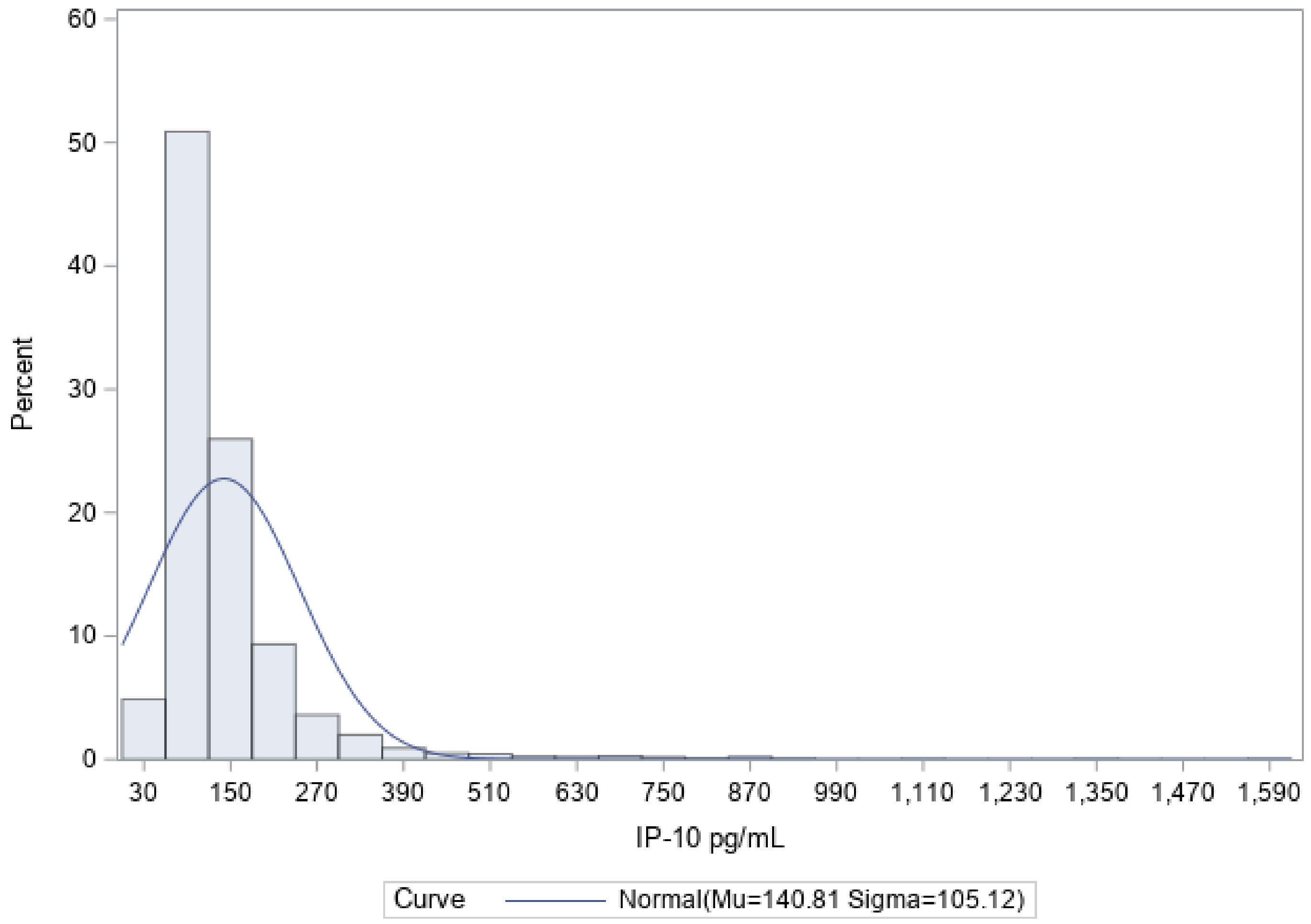

b. JHS, natural log transformed IP-10

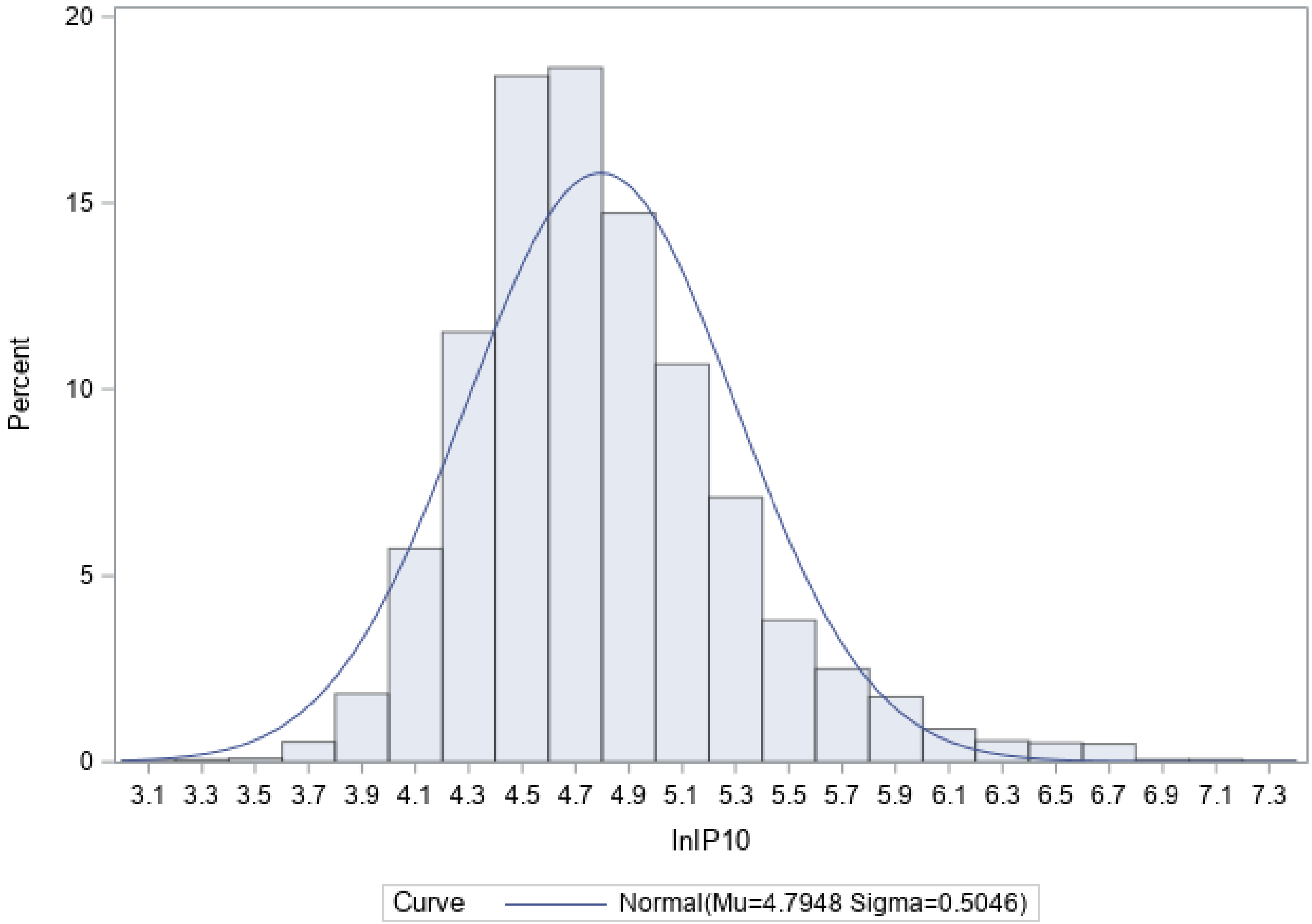

c. REGARDS cohort random sample only, IP-10 untransformed values in pg/mL

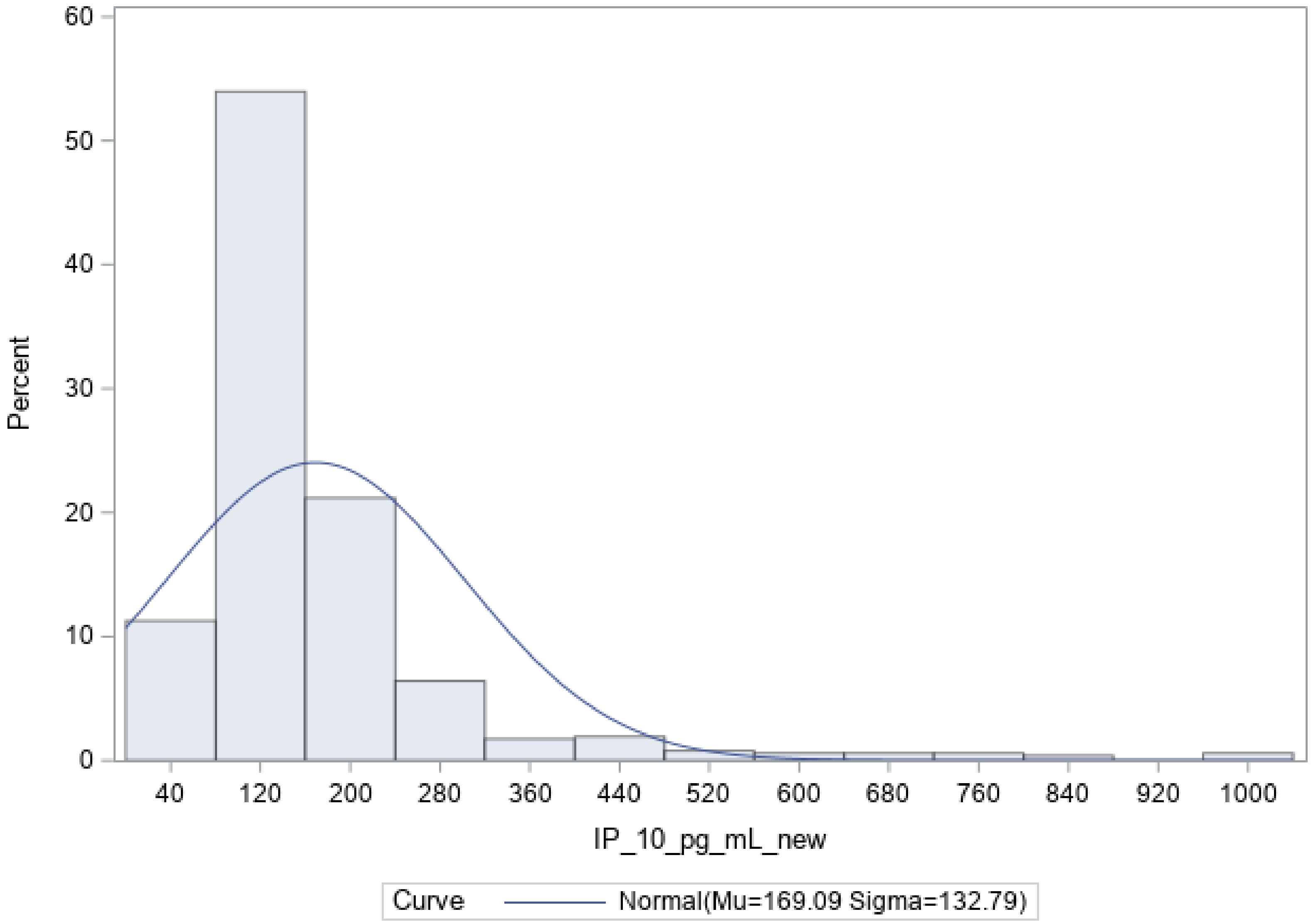

d. REGARDS cohort random sample only, natural log transformed IP-10

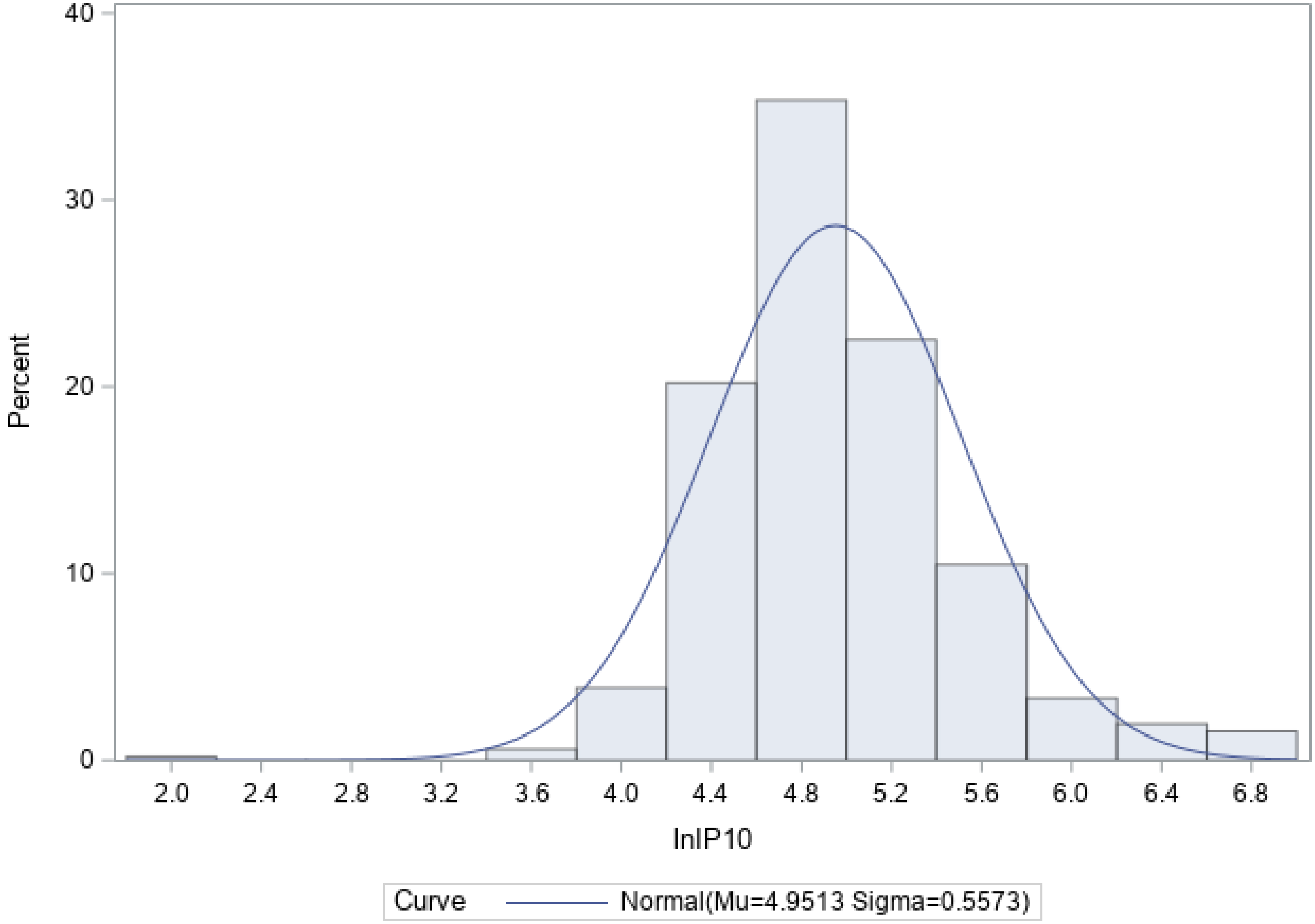

Supplement: S1 Fig — (PDF) [file pone.0231013.s001.pdf]
