# Supplementary material for: Interferon gamma-induced protein 10 (IP-10) and cardiovascular disease in African Americans
Source: PLoS One. 2020 Apr 2;15(4):e0231013. doi: 10.1371/journal.pone.0231013 (PMC7117698; doi:10.1371/journal.pone.0231013)

Supplementary Figure 2: Kaplan-Meier plots for IP-10 quartiles, unadjusted for covariates.

a. JHS, Mortality

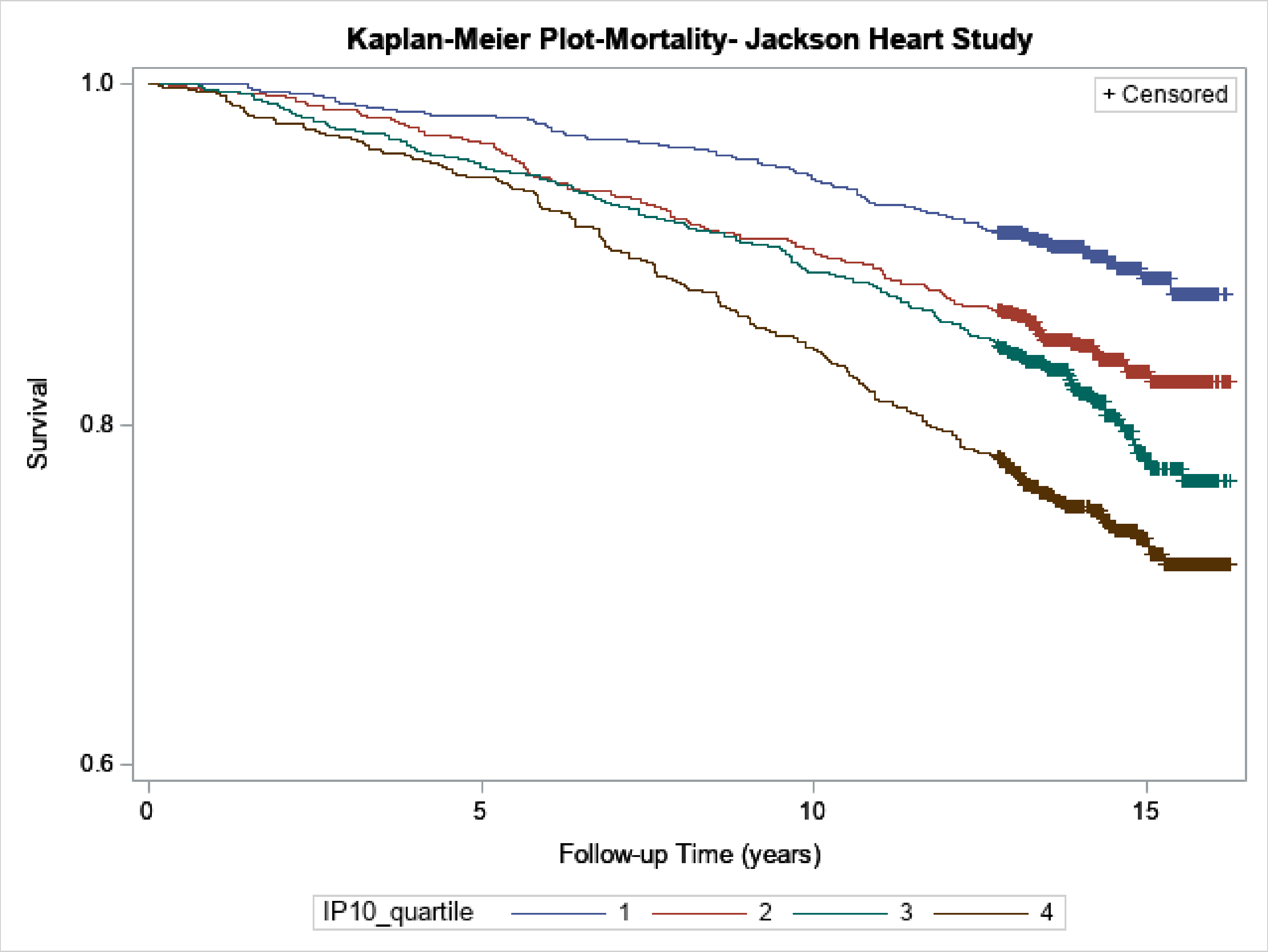

b. JHS, Stroke

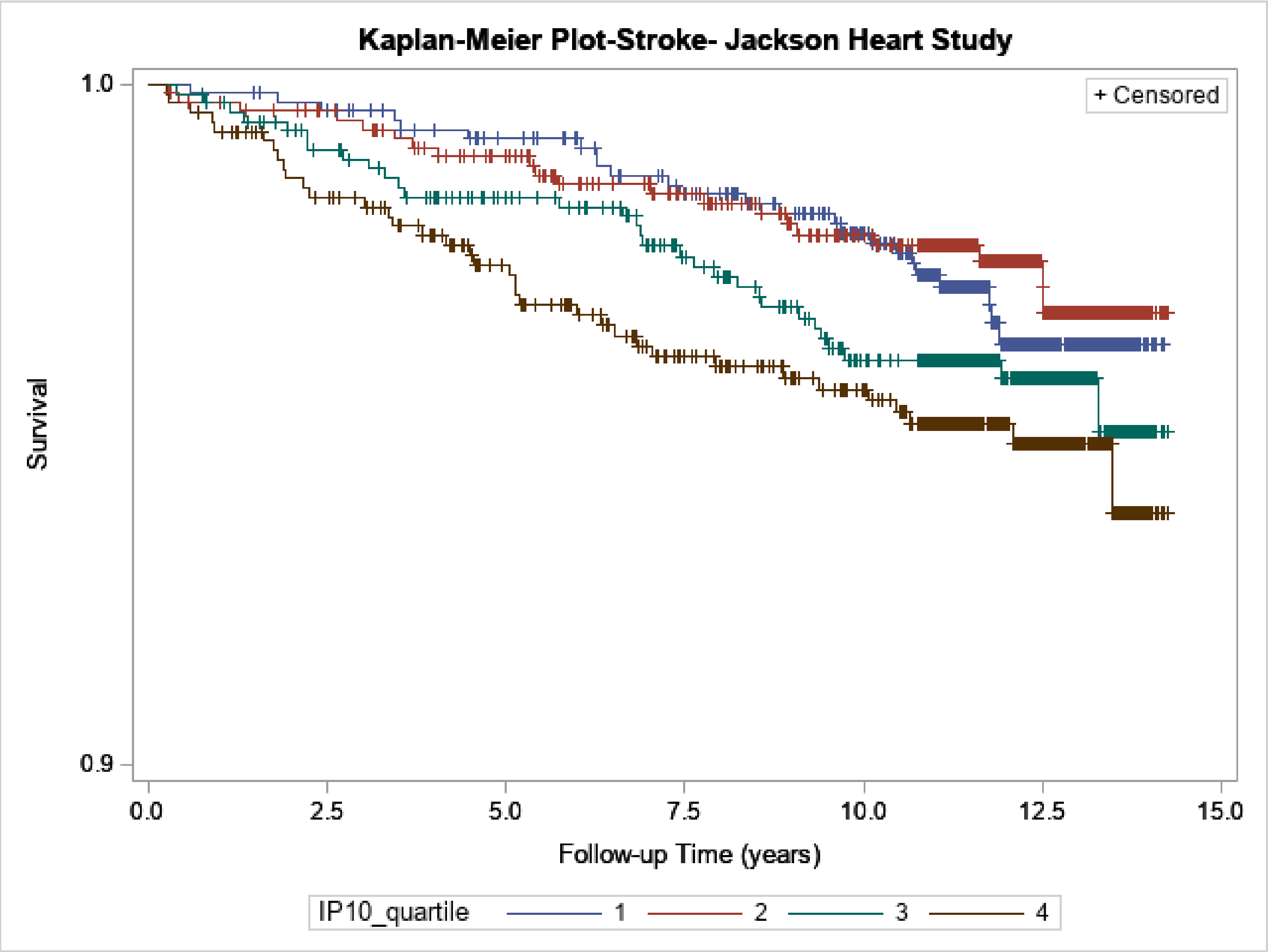
Kaplan-Meier Plot-Stroke- Jackson Heart Study
+ Censored
1.0
0.9
Survival
0.0
2.5
5.0
7.5
10.0
12.5
15.0
Follow-up Time (years)
IP10_quartile
1
2
3
4

c. JHS, CHD

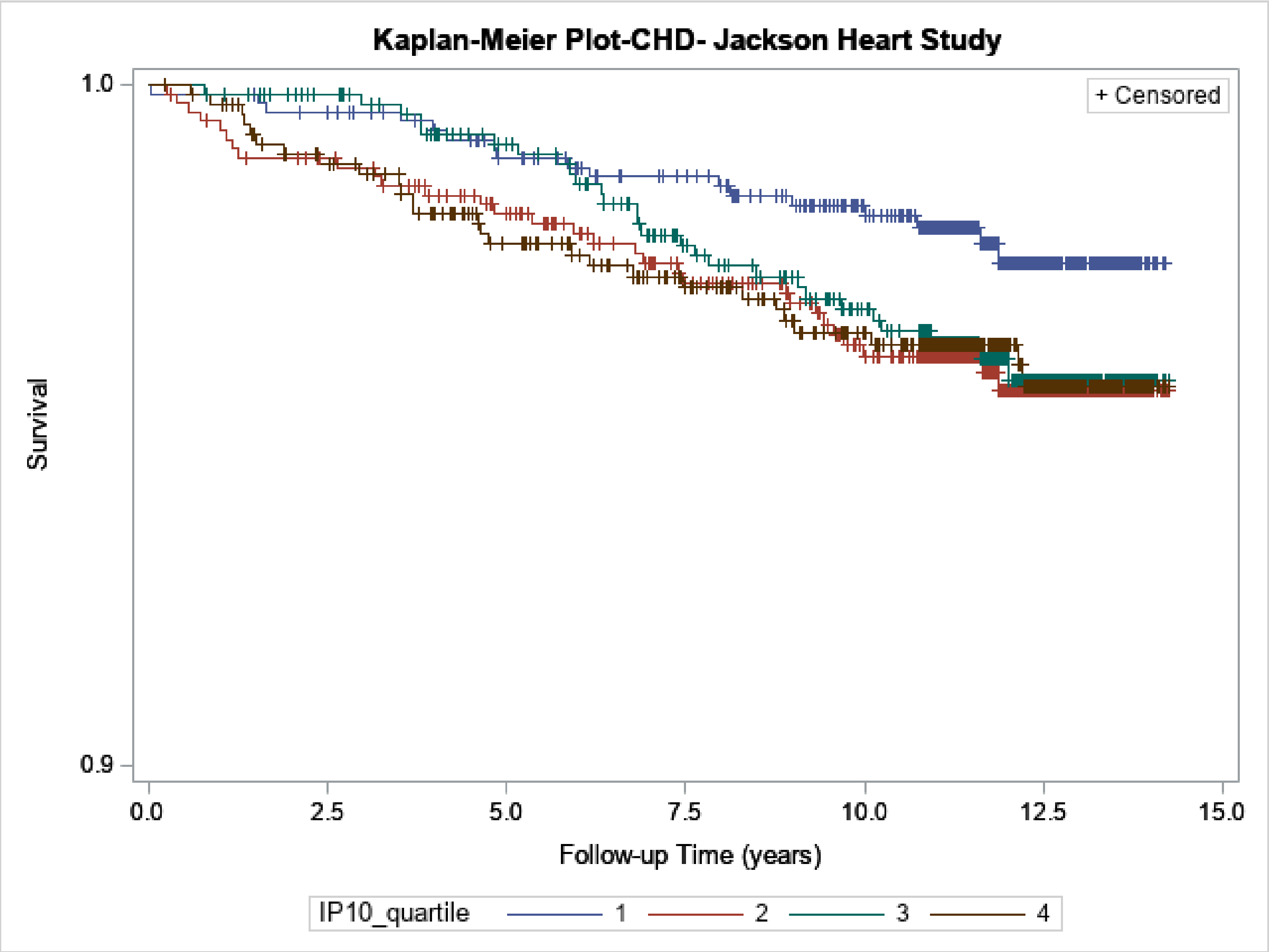

Kaplan-Meier Plot-CHD- Jackson Heart Study
+ Censored
1.0
0.9
Survival
0.0
2.5
5.0
7.5
10.0
12.5
15.0
Follow-up Time (years)
IP10_quartile
1
2
3
4

d. JHS, Heart failure

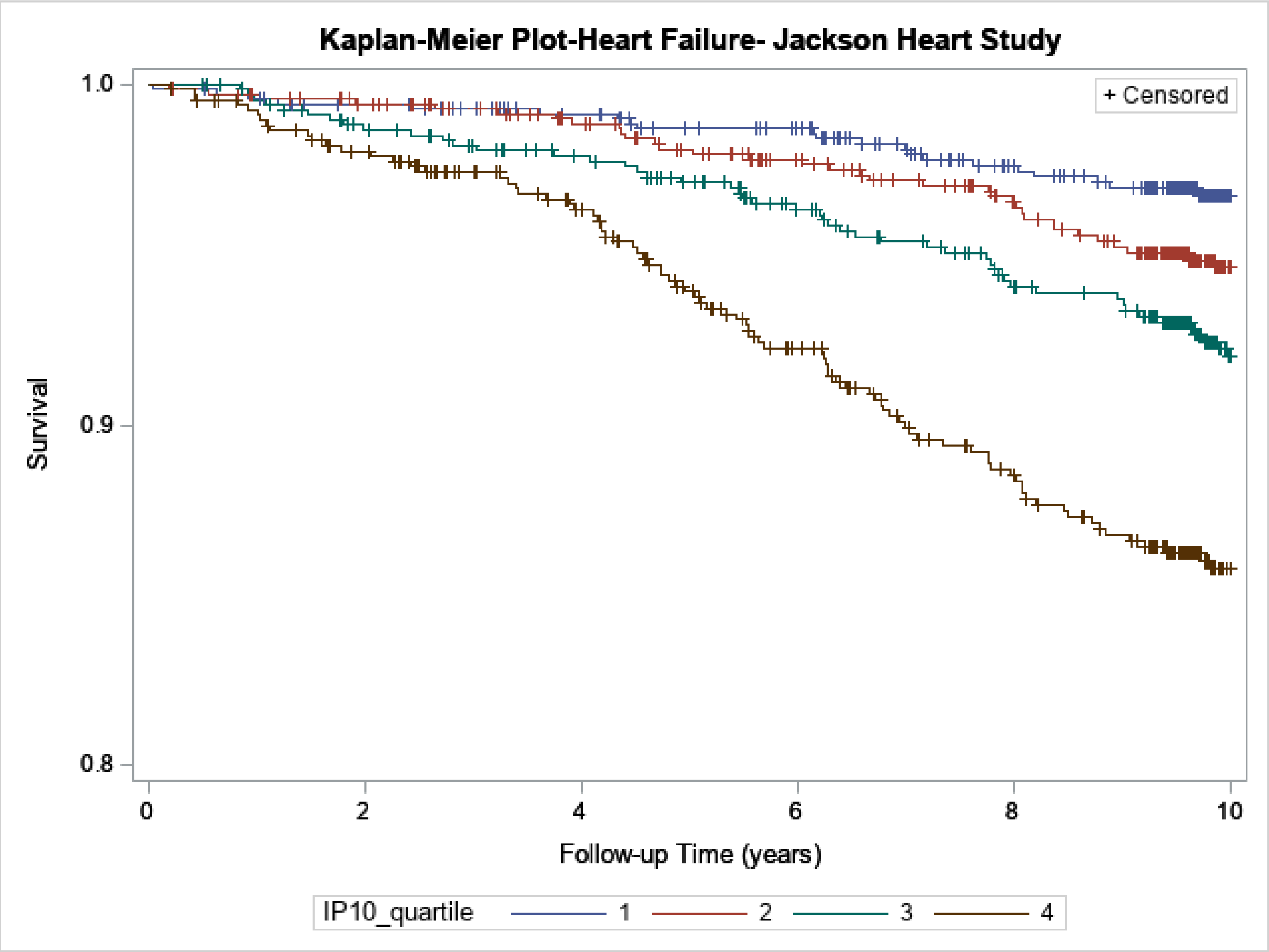

e. Mortality, REGARDS (cohort random sample)

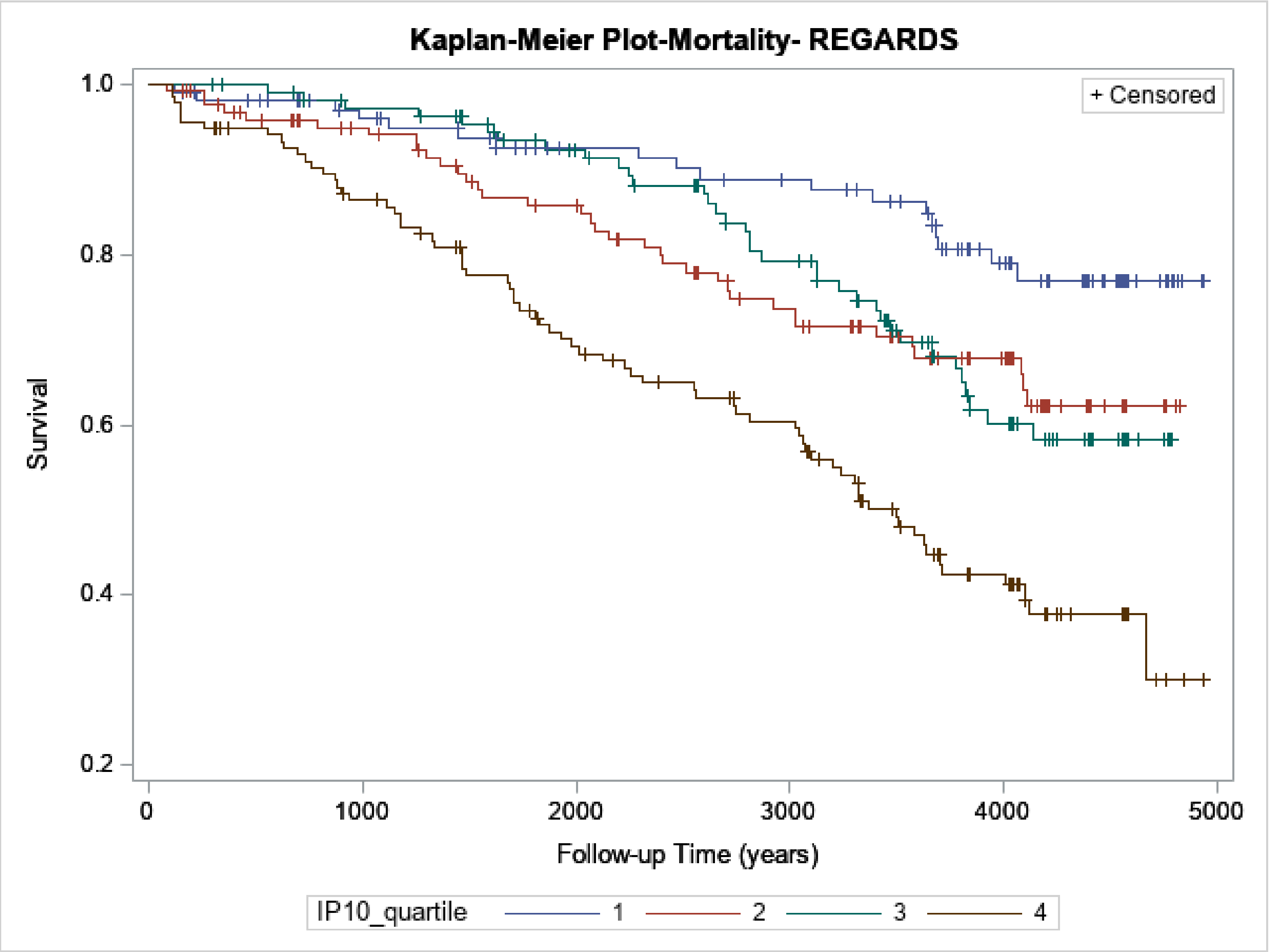

Supplement: S2 Fig — (PDF) [file pone.0231013.s002.pdf]
