# Supplementary material for: Interferon gamma-induced protein 10 (IP-10) and cardiovascular disease in African Americans
Source: PLoS One. 2020 Apr 2;15(4):e0231013. doi: 10.1371/journal.pone.0231013 (PMC7117698; doi:10.1371/journal.pone.0231013)
Supplement: S1 Table — Each quartile is reported in reference to quartile 1. Trend across quartiles is also reported. (PDF) [file pone.0231013.s003.pdf]

Supplementary Table 1: Association of IP-10 quartiles with mortality and incident cardiovascular disease events in JHS and REGARDS. Each quartile is reported in reference to quartile 1. Trend across quartiles is also reported.

			JHS				REGARDS			
			**Q2**	**Q3**	**Q4**	**Trend**	**Q2**	**Q3**	**Q4**	**Trend**
**Coronary Heart Disease**		**Events/N**	101/2906				466/868			
	**Model 1**	**HR (95% CI)**	1.51 (0.82, 2.78)	1.24 (0.65, 2.35)	1.08 (0.56, 2.10)	0.98 (0.82, 1.18)	1.43 (0.89, 2.30)	1.00 (0.62, 1.61)	1.59 (0.96, 2.63)	1.10 (0.94,1.29)
		**p-value**	0.19	0.51	0.82	0.85	0.14	1	0.07	0.22
	**Model 2**	**HR (95% CI)**	1.51 (0.82, 2.80)	1.30 (0.68, 2.50)	1.12 (0.58, 2.16)	1.00 (0.83, 1.19)	1.60 (0.95, 2.70)	1.02 (0.60, 1.72)	1.69 (0.94, 3.03)	1.10 (0.92,1.32)
		**p-value**	0.19	0.42	0.73	0.97	0.08	0.95	0.08	0.28
	**Model 3**	**HR (95% CI)**	1.52 (0.82, 2.81)	1.32 (0.69, 2.52)	1.12 (0.58, 2.16)	1.00 (0.83, 1.19)	1.68 (0.98, 2.88)	0.99 (0.57, 1.71)	1.75 (0.96, 3.19)	1.10 (0.92,1.33)
		**p-value**	0.19	0.41	0.73	0.97	0.06	0.97	0.07	0.29
**Stroke**		**Events/N**	110/2991				445/881			
	**Model 1**	**HR (95% CI)**	0.67 (0.38, 1.21)	0.90 (0.52, 1.55)	0.89 (0.51, 1.55)	1.00 (0.83, 1.21)	1.26 (0.78, 2.05)	0.94 (0.59, 1.52)	1.62 (0.98, 2.66)	1.12 (0.96,1.31)
		**p-value**	0.18	0.69	0.68	0.98	0.35	0.81	0.06	0.15
	**Model 2**	**HR (95% CI)**	0.67 (0.37, 1.20)	0.95 (0.55, 1.65)	0.97 (0.56, 1.68)	1.04 (0.86, 1.25)	1.35 (0.80, 2.28)	1.05 (0.63, 1.75)	1.73 (1.00, 3.00)	1.15 (0.97,1.36)
		**p-value**	0.17	0.86	0.91	0.70	0.26	0.85	0.05	0.11
	**Model 3**	**HR (95% CI)**	0.66 (0.36, 1.20)	0.99 (0.57, 1.71)	0.98 (0.57, 1.68)	1.05 (0.87, 1.26)	1.41 (0.83, 2.40)	0.99 (0.59, 1.67)	1.81 (1.03, 3.16)	1.15 (0.97,1.37)

		p-value	0.17	0.97	0.94	0.64	0.20	0.96	0.04	0.12
All-Cause Mortality		Events/N	559/3173				160/475			
	Model 1	HR (95% CI)	1.26 (0.96, 1.67)	1.29 (0.98, 1.70)	1.41 (1.08, 1.85)	1.1 (1.02, 1.19)	1.39 (0.78, 2.48)	1.19 (0.65, 2.15)	2.16 (1.24, 3.78)	1.27 (1.08,1.50)
		p-value	0.10	0.07	0.01	0.02	0.27	0.58	0.01	3.64×10^{-3}
	Model 2	HR (95% CI)	1.30 (0.98, 1.71)	1.33 (1.01, 1.75)	1.50 (1.14, 1.96)	1.12 (1.04, 1.22)	1.31 (0.73, 2.36)	1.19 (0.65, 2.19)	2.17 (1.23, 3.84)	1.29 (1.09,1.53)
		p-value	0.07	0.04	3.46×10^{-3}	0.01	0.37	0.57	0.01	2.61×10^{-3}
	Model 3	HR (95% CI)	1.30 (0.98, 1.72)	1.35 (1.03, 1.78)	1.50 (1.15, 1.96)	1.12 (1.04, 1.22)	1.37 (0.76, 2.48)	1.19 (0.65, 2.18)	2.16 (1.22, 3.82)	1.27 (1.08,1.51)
		p-value	0.07	0.03	2.98×10^{-3}	4.39×10^{-3}	0.30	0.58	0.01	4.41×10^{-3}
Heart Failure		Events/N	190/2756							
	Model 1	HR (95% CI)	1.37 (0.80, 2.36)	1.79 (1.07, 3.00)	2.76 (1.67, 4.54)	1.41 (1.21, 1.64)				
		p-value	0.25	0.03	6.89×10^{-5}	8.85×10^{-6}				
	Model 2	HR (95% CI)	1.41 (0.82, 2.44)	1.79 (1.07, 3.01)	2.74 (1.66, 4.52)	1.40 (1.20, 1.62)				
		p-value	0.22	0.03	8.30×10^{-5}	1.30×10^{-5}				
	Model 3	HR (95% CI)	1.41 (0.82, 2.44)	1.79 (1.07, 3.01)	2.74 (1.66, 4.52)	1.40 (1.20, 1.63)				
		p-value	0.22	0.03	8.46×10^{-5}	1.33×10^{-5}				

*Hazard ratios (HR) and 95% confidence intervals (CIs) are reported for Only individuals with complete covariates for all models are included. REGARDS did not have an adequate number of heart failure cases in the case-cohort study for analysis.

Model 1: Adjusted for age, sex (with additional adjustment for region in REGARDS)

Model 2: Model 1 + BMI, blood pressure medications, type 2 diabetes, systolic blood pressure, total cholesterol, high-density lipoprotein cholesterol, current smoking

Model 3: Model 2 + C-reactive protein
