# Supplementary material for: Interferon gamma-induced protein 10 (IP-10) and cardiovascular disease in African Americans
Source: PLoS One. 2020 Apr 2;15(4):e0231013. doi: 10.1371/journal.pone.0231013 (PMC7117698; doi:10.1371/journal.pone.0231013)
Supplement: S2 Table — (PDF) [file pone.0231013.s004.pdf]

Supplementary Table 2: Additional adjustment for the potential confounders kidney function (as assessed using estimated glomerular filtration rate (eGFR) calculated using the CKD-EPI equation) and brain natriuretic peptide (BNP) in the JHS heart failure models, as well as potential mediator left ventricular hypertrophy.

			JHS				
			**Q2**	**Q3**	**Q4**	**Trend**	**Per SD**
**Heart Failure**	**Model 4**	**Events/N**	187/2688				
		**HR (95% CI)**	1.34 (0.78, 2.32)	1.65 (0.98, 2.78)	2.52 (1.52, 4.20)	1.37 (1.17, 1.60)	1.21 (1.06, 1.38)
		**p-value**	0.29	0.06	3.75×10^{-4}	9.10×10^{-5}	6.2×10^{-3}
	**Model 5**	**Events/N**	190/2756				
		**HR (95% CI)**	1.35 (0.78, 2.33)	1.70 (1.01, 2.85)	2.51 (1.51, 4.19)	1.36 (1.17, 1.59)	1.23 (1.08, 1.39)
		**p-value**	0.28	0.05	4.19×10^{-4}	8.13×10^{-5}	2.1×10^{-3}
	**Model 6**	**Events/N**	187/2688				
		**HR (95% CI)**	1.29 (0.74, 2.23)	1.55 (0.91, 2.61)	2.33 (1.38, 3.91)	1.33 (1.14, 1.56)	1.19 (1.03, 1.36)
		**p-value**	0.37	0.10	1.45×10^{-3}	4.01×10^{-4}	0.02
	**Model 7**	**Events/N**	113/1811				
		**HR (95% CI)**	1.33 (0.66, 2.66)	1.93 (0.99, 3.74)	2.72 (1.48, 4.99)	1.41 (1.17, 1.69)	1.22 (1.04, 1.43)
		**p-value**	0.43	0.05	1.27×10^{-3}	2.37×10^{-4}	0.01
	**Model 8**	**Events/N**	112/1764				
		**HR (95% CI)**	1.23 (0.60, 2.50)	1.63 (0.82, 3.24)	2.42 (1.29, 4.54)	1.36 (1.13, 1.65)	1.15 (0.97, 1.37)
		**p-value**	0.58	0.16	5.76×10^{-3}	1.55×10^{-3}	0.11

* All models in this table adjusted for age, sex, BMI, blood pressure medications, type 2 diabetes, systolic blood pressure, total cholesterol, high-density lipoprotein cholesterol, current smoking, C-reactive protein

Model 4: Additional adjustment for BNP.

Model 5: Additional adjustment for eGFR.

Model 6: Additional adjustment for BNP and eGFR.

Model 7: Additional adjustment for LVH.

Model 8: Additional adjustment for BNP, LVH, and eGFR.
